# Perturbation-based balance training targeting both slip- and trip-induced falls among older adults: a randomized controlled trial

**DOI:** 10.1186/s12877-020-01605-9

**Published:** 2020-06-12

**Authors:** Leigh J. Allin, P. Gunnar Brolinson, Briana M. Beach, Sunwook Kim, Maury A. Nussbaum, Karen A. Roberto, Michael L. Madigan

**Affiliations:** 1 Department of Biomedical Engineering and Mechanics, Virginia Tech, Blacksburg, VA USA; 2 Edward Via College of Osteopathic Medicine, Blacksburg, VA USA; 3 Grado Department of Industrial and Systems Engineering, Virginia Tech, 250 Durham Hall (0118), 1145 Perry Street, Blacksburg, VA USA; 4 Institute for Society, Culture and Environment, Center for Gerontology, Virginia Tech, Blacksburg, VA USA

**Keywords:** Falls, Balance training, Step training, Perturbation-based balance training, Dynamic balance, Aging

## Abstract

**Background:**

Falls are the leading cause of injuries among older adults. Perturbation-based balance training (PBT) is an innovative approach to fall prevention that aims to improve the reactive balance response following perturbations such as slipping and tripping. Many of these PBT studies have targeted reactive balance after slipping *or* tripping, despite *both* contributing to a large proportion of older adult falls. The goal of this randomized controlled trial was to evaluate the effects of PBT targeting slipping *and* tripping on laboratory-induced slips and trips. To build upon prior work, the present study included: 1) a control group; 2) separate training and assessment sessions; 3) PBT methods potentially more amenable for use outside the lab compared to methods employed elsewhere, and 4) individualized training for older adult participants.

**Methods:**

Thirty-four community-dwelling, healthy older adults (61–75 years) were assigned to PBT or a control intervention using minimization. Using a parallel design, reactive balance (primary outcome) and fall incidence were assessed before and after four sessions of BRT or a control intervention involving general balance exercises. Assessments involved exposing participants to an unexpected laboratory-induced slip or trip. Reactive balance and fall incidence were compared between three mutually-exclusive groups: 1) baseline participants who experienced a slip (or trip) before either intervention, 2) post-control participants who experienced a slip (or trip) after the control intervention, and 3) post-PBT participants who experienced a slip (or trip) after PBT. Neither the participants nor investigators were blinded to group assignment.

**Results:**

All 34 participants completed all four sessions of their assigned intervention, and all 34 participants were analyzed. Regarding slips, several measures of reactive balance were improved among post-PBT participants when compared to baseline participants or post-control participants, and fall incidence among post-PBT participants (18%) was lower than among baseline participants (80%). Regarding trips, neither reactive balance nor fall incidence differed between groups.

**Conclusions:**

PBT targeting slipping and tripping improved reactive balance and fall incidence after laboratory-induced slips. Improvements were not observed after laboratory-induced trips. The disparity in efficacy between slips and trip may have resulted from differences in dosage and specificity between slip and trip training.

**Trial registration:**

**Name of Clinical Trial Registry:** clinicaltrials.gov

Trial Registration number: NCT04308239.

**Date of Registration:** March 13, 2020 (retrospectively registered).

## Background

Falls are the leading cause of injuries among adults age 65 and older United States [1]. In 2014, for example, 29 million falls were reported among older adults, resulting in 7 million injuries [2] and costing $50 billion [3]. Moreover, the number of annual falls among older adults continues to grow at a rate that is faster than the growth rate of the older adult population [4]. These statistics, despite years of effort toward fall prevention, highlight the need for more effective methods to reduce falls.

Perturbation-based balance training (PBT) is a promising new approach to fall prevention that involves training the neuromuscular response to postural perturbations [5–7]. PBT, when tailored to mimic common fall scenarios such as slipping or tripping, is a form of task-specific training that leverages motor learning principles by allowing participants to practice reactive responses in a safe, controlled setting [5]. Slips commonly occur at heel strike when the friction required for walking exceeds the friction available between the foot and floor [8], and typically result in a backwards loss of balance [9]. Examples of PBT for slipping have involved repeated exposure to slips while walking over a sliding platform [6], or a structured step-training regimen onto a low-friction surface [10]. These types of PBT have elicited slip-reducing proactive gait adaptations, and improved recovery rates and reactive balance after laboratory-induced slips. Trips commonly occur when an obstruction impedes the forward motion of the swing leg during gait, and typically result in a forward loss of balance [11]. Examples of PBT for tripping have involved repeated exposure to trips while walking over ground [12], using a cable system to impede forward foot motion while walking on a treadmill [13], or simulated trips while standing on a specialized treadmill [5, 14]. These types of PBT have elicited improved kinematics and fall rates after laboratory-induced trips [5, 15], and reduced trip-induced falls outside of the laboratory [16].

Nearly all prior PBT studies have targeted either slips or trips, but not both, despite both types of perturbations being responsible for a substantial percentage of falls among older adults [17]. Only a few studies we are aware of have incorporated slip and trip training into the same PBT regimen. Bhatt et al. [18] had young adult participants complete a single testing session involving repeated laboratory-induced slips and trips while walking along a 7-m-long specialized walkway. Over the repeated perturbations, participants developed gait adaptations involving reduced step length and higher toe clearance to reduce the risk of slips and trips, and improved their reactive balance after slips and trips by increasing center-of-mass (COM) state stability and minimum hip height [18]. Three similar studies from another research group also had participants complete a single training/testing session involving repeated laboratory-induced slips and trips while walking along a specialized 10-m-long walkway [19–21]. Over the course of this latter training, participants generally improved their reactive balance and reduced fall incidence after slipping, and (albeit inconsistently) their reactive balance after tripping. These latter three studies constrained gait to discourage changes in gait step length and frequency during training because any gait adaptations such as these can confound reactive balance measurements, and hence the results may not generalize to less predictable falls in daily life [20]. Overall, these studies support the continued development of PBT to simultaneously address slip- and trip-induced falls.

The goal of this study was to evaluate the effects of PBT targeting slipping *and* tripping on laboratory-induced slips and trips. In an effort to build upon prior studies that had a similar goal, the present study included: 1) a control group receiving an alternative balance training intervention; 2) separate training and assessment sessions; 3) alternative PBT methods that may be more amenable to implementation outside the lab compared to methods used elsewhere, and 4) training individualized to each older adult participant’s capabilities to reduce drop-out [21] and increase efficacy. We hypothesized that participants completing PBT would exhibit a lower peak slip speed after a laboratory-induced slip, compared to participants before intervention or after a control intervention. We also hypothesized that participants completing PBT would exhibit a smaller trunk angle at touchdown of the first recovery step after a laboratory-induced trip, compared to participants before intervention or after a control intervention. Our results were intended to contribute to knowledge regarding the efficacy of PBT.

## Methods

Participants were 34 community-dwelling adults (61–75 years, 19 female) recruited from the university and local community using participant lists from the Virginia Tech Center for Gerontology, email listservs, posted fliers, and visits to local community organizations. Participants were initially screened by phone to exclude those who: 1) smoked, 2) were in physical therapy, 3) had a self-reported fragility fracture within the last 10 years, 4) had an acute lower extremity injury within the last 3 months, 5) had lower extremity surgery within the last 6 months, 6) had an ankle arthroplasty, or 7) had a knee or hip arthroplasty within the last 12 months. Participants were also required to pass a medical history and screening administered by a physician that excluded participants with osteoporosis of the lumbar spine or proximal femur as assessed by Dual Energy X-ray Absorptiometry (Lunar iDXA, GE Healthcare, Chicago, IL), or any unstable or progressive medical conditions that could contribute to imbalance or falls. Recruitment started in November 2018, and all training and testing was completed in June 2019. The study adheres to CONSORT guidelines, and was approved by the Virginia Tech Institutional Review Board. All participants provided written informed consent prior to participation.

**Fig. 1:**
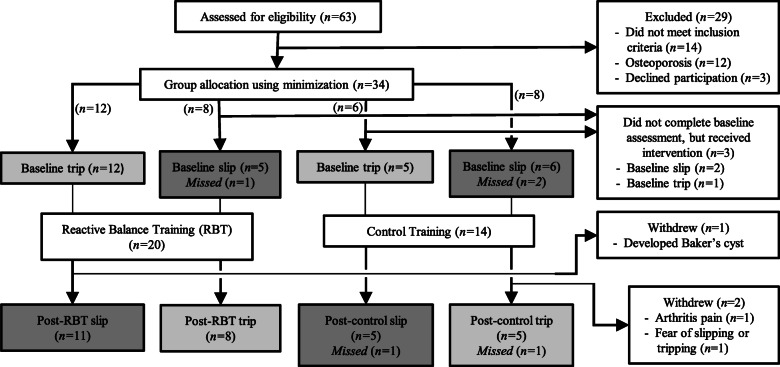
Flow diagram showing participant enrollment, allocation, and exclusion. Groups shaded in medium gray were included in the slip comparisons (with baseline slips pooled into one group), while groups shaded in light gray were included in the trip comparisons (with baseline trips pooled into one group). Missed slips or trips are described in the text, and resulted in missing data

A two-group, pretest-posttest parallel design was employed to evaluate the effects of PBT on laboratory-induced slips and trips (Fig. 1). Participants were first assigned to either the PBT or control intervention using minimization [22] to balance groups with respect to age, sex, and physical activity level as quantified by the International Physical Activity Questionnaire [23]. Although a 1:1 allocation ratio between interventions was planned, a clerical error resulted in an actual allocation ratio of approximately 3:2. During a baseline assessment session, participants were exposed to an unexpected laboratory-induced slip or trip based upon random assignment. Starting approximately 1 week later, participants completed four sessions of their assigned intervention with these sessions scheduled twice a week for 2 weeks. The post-intervention assessment occurred the following week during which participants were exposed to the other perturbation (slip or trip) that they did not experience during the baseline assessment. As such, each participant was exposed to one laboratory-induced slip and one laboratory-induced trip during the study, with one occurring during the baseline assessment before intervention, and the other during the post-assessment after intervention. This design was selected to evaluate gait and reactive balance prior to any training intervention, and to avoid any unintended training effects induced by exposing participants to the same perturbation more than once. All sessions took place in a research lab, and were administered by the same investigator who made all group and perturbation assignments.

Separate statistical analyses were completed for slips and trips, with each analysis involving comparisons between three groups: 1) baseline participants exposed to a slip (or trip) during the baseline assessment, 2) post-control participants exposed to a slip (or trip) after the control intervention, and 3) post-PBT participants exposed to a slip (or trip) after PBT. In each analysis (slips or trips), the three groups involved were mutually exclusive. Significant differences between baseline participants and post-PBT participants would provide evidence of efficacy based upon PBT-induced changes in reactive balance and fall incidence, while differences between post-control participants and post-PBT participants would provide stronger evidence for efficacy based upon PBT-induced changes compared to a control intervention.

**Fig. 2:**
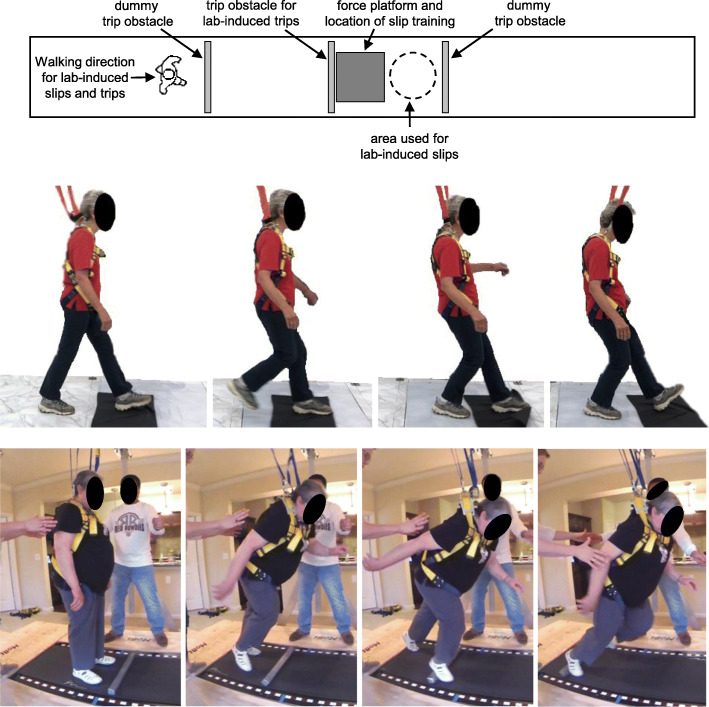
Top: Top-down view of walkway used for PBT slip training and lab-induced slips and trips. Middle: Time-lapse photos of PBT for slip training. Bottom: Time-lapse photos of PBT for trip training. A harness was worn during slip and trip training, and a spotter was also nearby for instruction and support. During PBT for trip training, a light-weight foam block was placed in front of the participant’s feet prior to each perturbation to promote a stepping response over an obstacle, as is necessary during actual trip recovery

Baseline, post-control, and post-PBT assessments included a battery of tests. Clinical tests to assess balance and mobility at baseline included the timed up-and-go test [24], single-leg stance time [25], the performance-oriented mobility assessment (POMA) [26], and balance confidence assessed using the falls efficacy scale [27]. Participants wore their own walking or athletic shoes for these assessments. Reactive balance was then assessed in response to an unexpected laboratory-induced slip or trip while walking. Participants were instructed to walk along a 12-m level walkway (Fig. 2) at a self-selected pace “as if you have somewhere to go” and, if slipped or tripped, to recover balance and continue walking. These same instructions were used when measuring unperturbed gait characteristics and responses to slips or trips. After initial walking trials to determine a self-selected gait speed, subsequent trials were constrained to within 0.1 m/s of this speed using a motion capture system to track a marker on the sacrum to measure mean gait speed and verbal feedback after each trial as needed to walk slightly faster or slower. Walking trials with speeds outside of this range were discarded and repeated. After a minimum of 10 trials, participants were exposed to a slip or trip using methods described earlier [9, 28, 29]. In brief, slips were induced by spreading a thin layer of vegetable oil over a 0.9 × 0.9 m section of the walkway while participants were facing away, and slips occurred when the heel of the dominant foot contacted the oil. Trips were induced using a tripping obstacle that was initially concealed and level with the walkway. Upon proper placement of the stance foot relative to the obstacle while walking, the obstacle was activated and quickly rose to a height of 8.6 cm. A trip occurred when the dominant foot contacted the obstacle near the middle of the subsequent swing phase.

To minimize anticipation to a slip or trip: 1) participants were asked to face away from the walkway for a fixed time prior to all trials while the investigators purposefully made misleading experimental-relevant noises; 2) two “dummy” trip obstacles were integrated in the walkway to prevent anticipation of trip location; and 3) participants were only slipped and tripped once each so no precedent could be used to predict an up-coming perturbation. After the protocol, all participants indicated being unable to anticipate the timing and location of the perturbations. All participants wore the same model of rubber-soled shoes during slip and trip assessments. During all trials, participants wore a fall protection harness affixed to a ceiling mounted track that spanned the length of the walkway. The length of the harness lanyard was set so that the distance between the participant’s knee and the floor when kneeling in the harness was approximately 20 cm.

Both PBT and control interventions involved four training sessions conducted twice per week for 2 weeks and in groups of 1–2 participants. Each session lasted approximately 0.5 to 1.0 h with an active training time for each participant of approximately 30 min. Training for each participant began with a five-minute warm-up of walking on a treadmill and light stretching.

PBT involved both slip and trip training. One of the first two training sessions involved only slip training, while the other involved only trip training, with the order of presentation counterbalanced within each participant group. The subsequent two training sessions involved equal proportions of both slip and trip training. To prevent falls, the fall protection harness was worn during all PBT sessions. Participants wore their own walking or athletic shoes during training.

PBT for slip training has been reported in detail elsewhere [10]. Briefly, participants repeatedly stepped onto a low-friction interface (nylon fabric placed over a 0.9 × 0.9 m polycarbonate sheet) while practicing controlling/decelerating the slipping foot and properly positioning the non-slipping foot under the pelvis by stepping (Fig. 2). Both of these actions are critical to prevent a fall after slipping while walking [9]. Training was individualized to participant capability as evaluated qualitatively and visually by the investigator (LJA) administering training. Training began with a single step onto the fabric to induce short, slow, self-initiated slips, and progressed to walking several steps then onto the fabric to induce longer, faster slips as when slipping while walking. The limb performing the initial step onto the fabric was specified and varied by the trainer in an effort to train the responses for both feet. Participants completed 60–80 slip-like perturbations during each training session dedicated solely to slip training, and 30–40 slip-like perturbations during each training session that involved slip and trip training.

PBT for trip training has also been reported elsewhere [5, 14]. Briefly, participants stood on a modified treadmill. At a random time, the treadmill belt was quickly accelerated posteriorly to an investigator-selected speed within approximately 40 ms to elicit a forward loss of balance similar to a trip while walking (Fig. 2). Participants attempted to recover their balance by stepping, and establish a stable gait on the treadmill, after which the treadmill speed was gradually slowed to zero to prepare for the next trial. Perturbation speeds were varied pseudo-randomly, individualized to participant capability as evaluated qualitatively and visually by the investigator during training, and progressively increased as performance improved. To prevent participants from anticipating forward losses of balance, backward losses of balance (induced by anterior belt accelerations) were pseudo-randomly interspersed throughout training. As during slip training, the initial stepping limb was specified and varied by the trainer. Participants completed 30 trip-like perturbations during each training session dedicated solely to trip training, and 20 trip-like perturbations during each training session that involved slip and trip training.

The control intervention involved general balance exercises adapted from the Otago Exercise program [30], which has been shown to reduce fall risk among community-dwelling older adults [30]. We chose Otago, rather than another or no control intervention, to increase the level of evidence required to demonstrate efficacy, and because, similar to the PBT employed here, it was designed to be performed individually or in small groups. Briefly, all four sessions involved balance exercises and strength exercises using ankle weights, and were progressively increased as performance improved by increasing ankle weights or the difficulty of the balance exercises (e.g., not holding onto a wall or support).

Body kinematics, ground reaction forces, and force applied to the harness were measured during gait, slip, and trip trials of baseline and post-intervention assessments. The three-dimensional kinematics of 35 reflective markers and five rigid marker clusters were sampled at 120 Hz using a 13-camera motion capture system (Qualisys North America, Inc., Buffalo Grove, IL), then low-pass filtered at 12 Hz (fourth-order, zero-phase-lag Butterworth filter). Ground reaction forces were sampled at 1200 Hz using a 0.9 × 0.9 m force platform (Bertec, Columbus, OH) embedded in the walkway. Force applied to the safety harness was sampled at 1200 Hz using a uniaxial load cell (Cooper Instruments, Warrenton, VA). Force platform and load cell signals were low-pass filtered at 40 Hz (fourth-order, zero-phase-lag Butterworth filter).

Gait characteristics were measured during baseline and post-intervention assessments. These characteristics included *gait speed* (mean anteroposterior, or AP, speed of a sacral marker), *step length* (AP distance between lateral malleolus markers during consecutive stance phases), *minimum toe clearance* (local minimum of the vertical coordinate of a marker placed on the dorsal surface of the tip of the shoe during the swing phase of gait, measured relative to the vertical position at toe-off) [31], and *required coefficient of friction* (RCOF; maximum ratio of the rearward resultant shear force and vertical force during stance near heel contact) [32]. The latter two measures are indicators of risk of tripping and slipping, respectively, and were measured along with gait speed and step length to identify possible fall-relevant gait adaptations.

Several slip-related measures were obtained during baseline and post-intervention assessments. Critical temporal events were: *slip onset* (when the heel contacted the slippery surface), *touchdown* (when the non-slipping foot first contacted floor as a part of a reactive step following slip onset), and *slip end* (when the slipping heel either came to a stop, displaced vertically from the walkway, or the harness supported more than 50% body weight). The primary outcome measure of slip-related reactive balance was *peak slip speed* (the maximum resultant speed of the slipping heel from slip onset to slip end), which is both a measure of slip severity and the neuromuscular response to slipping. Secondary outcome measures included: *slip distance* (the total distance traveled by the slipping heel from slip onset to slip end, and is both a measure of slip severity and the neuromuscular response to slipping), *non-slipping toe to COM at touchdown* (AP distance between a marker on the toe of the non-slipping foot and the COM), *minimum hip height* (minimum height of the midpoint between the hip joint centers [33] following slip onset, expressed as percent hip height during standing), *COM velocity relative to base of support* (AP speed of COM relative to the slipping heel), and *margin of stability at touchdown* (shortest AP distance between the heel marker on the non-slipping foot that executed the initial recovery step, and the extrapolated COM) [34]. When the extrapolated COM was anterior to the heel marker (i.e. within the base of support), the margin of stability was positive; when posterior to this boundary (i.e. outside of the base of support), the margin of stability was negative. The body COM was calculated using a method described by [35].

Several trip-related measures were obtained during baseline and post-intervention assessments. Critical temporal events were: *trip onset* (when the leading edge of the tripped foot contacted the trip obstacle as indicated by the AP acceleration of a marker at the anterior tip of the shoe), and *touchdown* (when the initial recovery step over the obstacle contacted the floor as indicated by the vertical acceleration on a marker on the lateral malleolus). The primary outcome measure of trip-related reactive balance was *trunk angle at touchdown* (angle of a line from vertical connecting the midpoint between the hip joint centers and the midpoint between markers on each acromion process). Secondary outcome measures included: *recovery step length* (distance between a marker on the lateral malleolus of the stance limb and a marker on the lateral malleolus of the stepping foot at touchdown), *minimum hip height* (following trip onset), *stepping strategy* (elevating or lowering) [36], and *margin of stability at touchdown* (shortest AP distance between the toe marker on the foot that executed the initial recovery step over the obstacle, and the extrapolated COM). When the extrapolated COM was posterior to the toe marker (i.e. within the base of support), the margin of stability was positive; when anterior to this boundary (i.e. outside of the base of support), the margin of stability was negative.

Additional secondary outcome measures were fall incidences after slipping and tripping. Each slip (or trip) was classified as either a recovery, fall, harness-assist, or miss based on the force applied to the harness and video review of the event [37]. A recovery occurred if a 1-s moving average did not exceed 5% body weight during the trial. A fall occurred if a participant was fully and continuously supported by the harness as observed from video. A harness-assisted trial occurred if a trial was neither a recovery nor a fall. Missed slips occurred if slip distance was less than 10 cm and mean slipping heel speed was less than 0.5 m/s, and generally resulted from the heel not contacting the contaminant. Missed trips occurred if the leading edge of the swing foot did not contact the tripping obstacle during mid-to-late swing phase, and resulted from improper triggering of the trip obstacle. Missed slips or trips were not repeated, and were not included in the analyses.

Welch’s analysis of variance was used to compare continuous measures between the three groups to accommodate heterogeneity of variances between groups. When one group exhibited no variability, the nonparametric Kruskal-Wallis test was used. Where relevant, pairwise comparisons were performed using the Games-Howell Test, or nonparametric Wilcoxon test. Fisher’s Exact test was used to compare fall incidence between the three groups, as well as other nominal measures. We estimated a necessary sample size of 36 participants (equally split between intervention groups) using PBT effect sizes reported elsewhere for clinically-relevant reductions in peak trunk angle after tripping (Cohen’s *d* = − 1.24) [19], and slip speed after slipping (*d* = − 1.59) [21]. These differences were considered clinically-relevant, based on the anchor-based method for determining minimal clinically important difference [38], given that they were comparable to effect sizes between falls and recoveries after laboratory-induced trips [39] and slips [9] reported elsewhere. Using G*Power [40] for an independent *t*-test, and specifying 80% power and a one-sided Type I error rate of 5%, a sample size of nine participants per group was necessary. Nine participants in the post-PBT and post-control groups for each of the slip and trip analyses resulted in 18 participants in the baseline group (Fig. 1), and thus a total of 36 participants. Only 34 participants started and completed testing due to funding deadlines. All statistical analyses were performed using JMP Pro 12 (SAS Institute Inc., Cary, NC) and a significance level of *p* ≤ .05 with no adjustment for multiple comparisons.

## Results

Thirty-one participants completed the baseline balance assessment, all 34 participants completed all four intervention sessions, and 31 participants completed the post-intervention balance assessment (three participants declined further participation after completing the intervention, but not for reasons that threatened PBT adherence).

### Comparisons between slip groups

**Fig. 3:**
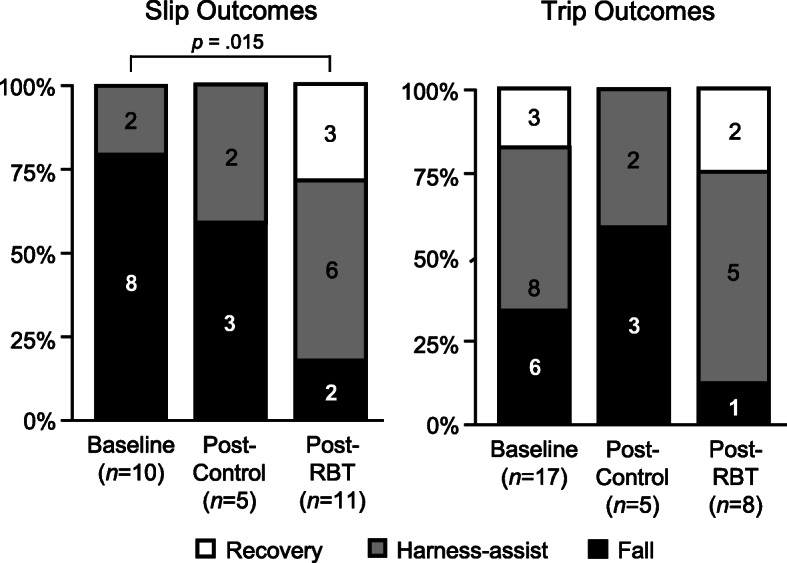
Outcomes of laboratory-induced slips (left) and trips (right) for all three participant groups. After slips, the incidence of falls (black) was 62% lower for post-PBT participants, compared to baseline participants (*p* = .027)

Participant characteristics did not differ between the three slip groups (Table 1). While gait characteristics also did not differ between the three slip groups (Table 2), several slip-related measures did. Peak slip speed, minimum hip height, margin of stability at touchdown of the non-slipping foot, and COM velocity relative to the base of support at touchdown of the non-slipping foot all exhibited between-group differences (Table 2). More specifically, peak slip speed among post-PBT participants was a mean of 0.57 m/s [95% CI = 0.05, 1.10] lower than among post-control participants (*p* = .034; Table 3). Minimum hip height among post-PBT participants was a mean of 5.6% [1.1, 10.1] higher than among baseline participants (*p* = .014; Table 3). Margin of stability at touchdown of the non-slipping foot among post-PBT participants was a mean of 11.1 cm [0.6, 21.7] larger than among baseline participants (*p* = .038; Table 3). Lastly, COM velocity relative to the slipping heel at touchdown of the non-slipping foot among post-PBT participants was a mean of 0.78 m/s [0.25, 1.31] lower than baseline participants (*p* = .005; Table 3), and among post-control participants was a mean of 0.50 m/s [0.01, 0.99] lower than among baseline participants (*p* = .047; Table 3). Fall incidence after slipping among post-PBT participants (18%) was lower than among baseline participants (80%; *p* = .027; Fig. 3). Additional statistical results and plots showing individual data points for continuous outcome measures are included in online Supplementary material.

**Table 1:** Participant characteristics for the three slip groups. Values are means (standard deviation)

	Baseline (*n* = 11)	Post-Control (*n* = 5)	Post-PBT (*n* = 11)	ANOVA *p*-value
Female/Male	6/5	3/2	5/6	
Age (years)	68.2 (3.5)	69.6 (5.3)	71.1 (3.3)	.203
Height (m)	1.71 (0.08)	1.67 (0.11)	1.68 (0.11)	.724
Mass (kg)	83.6 (15.4)	80.3 (16.4)	74.4 (16.4)	.441
IPAQ (MET minutes per week)	3355 (3845)	6384 (5083)	2371 (1815)	.268
Falls in the past year	0 (0%)	0 (0%)	1 (9%)	.483^K^
Timed-up-and-go (s)	8.4 (1.2)	7.6 (1.3)	7.7 (1.4)	.425
POMA (0–28 [best])	26.6 (1.1)	27.2 (0.8)	26.5 (1.0)	.415
Falls Efficacy Scale (10 [best] -100)	12.0 (4.5)	10.0 (0.0)	11.8 (3.1)	.332^K^

*Note*: *IPAQ* International Physical Activity Questionnaire, short form, *POMA* performance-oriented mobility assessment, ^K^ indicates Kruskal-Wallis Test was used

**Table 2:** Gait characteristics before slipping, and reactive balance measures in response to slipping, for the three slip groups. Values are means (standard deviation)

	Baseline (*n* = 11)	Post-Control (*n* = 5)	Post-PBT (*n* = 11)	ANOVA *p*-value
*Gait characteristics before slipping*				
Gait speed (m/s)	1.48 (0.11)	1.53 (0.13)	1.55 (0.11)	.314
Step length (%BH)	43.3 (0.3)	44.7 (0.4)	44.7 (0.2)	.530
Minimum toe clearance (mm)	17.0 (6.9)	26.8 (14.0)	22.1 (9.5)	.230
Required coefficient of friction	0.21 (0.02)	0.22 (0.03)	0.21 (0.02)	.756
*Reactive balance in response to slipping*				
Peak slip speed (m/s)	**2.80 (0.32)**	**3.15 (0.33)**	**2.58 (0.41)**	**.041**
Slip distance (cm)	80.8 (4.5)	74.2 (6.6)	71.4 (4.5)	.346
Non-slipping toe to COM at TD (%BH)	−5.8 (6.0)	−2.7 (6.8)	− 2.2 (9.2)	.498
Minimum hip height (%)	**80.6 (3.9)**	**82.0 (5.7)**	**86.2 (4.4)**	**.043**
Margin of stability at TD (cm)	**5.9 (4.8)**	**13.6 (6.8)**	**17.0 (12.3)**	**.027**
vCOM relative to BOS at TD (m/s)	**1.02 (0.23)**	**0.52 (0.32)**	**0.24 (0.62)**	**.004**

See Methods text for boundaries used to calculate margin of stability. A smaller positive vCOM relative to BOS indicates the slipping foot and COM are moving apart less quickly

*BH* body height, *COM* center of mass, *TD* touchdown of non-slipping foot after reactive stepping, *vCOM* anterior-posterior velocity of the COM, *BOS* base of support

Bold indicates statistically significant main effect of slip group. Minimum hip height is expressed as a percentage of standing hip height

**Table 3:** 95% confidence intervals of group differences for measures exhibiting statistically significant ANOVA results

	post-PBT minus post-control	post-PBT minus baseline	post-control minus baseline
Peak slip speed (m/s)	**−1.10, − 0.05**	− 0.615, 0.181	−0.151, 0.863
Minimum hip height (%)	−4.5, 12.9	**1.1, 10.1**	− 7.3, 10.2
Margin of stability at TD (cm)	−9.2, 16.2	**0.6, 21.7**	−2.8, 18.1
vCOM relative to BOS at TD (m/s)	− 0.900, 0.341	**−1.31, − 0.25**	**−0.99, − 0.01**

*COM* center of mass, *TD* touchdown of non-slipping foot after reactive stepping, *vCOM* anterior-posterior velocity of the COM, *BOS* base of support

Intervals that do not include zero are statistically significant and in bold. Minimum hip height is expressed as a percentage of standing hip height

### Comparisons between trip groups

Participant characteristics did not differ between the three trip groups (Table 4). Gait characteristics, including the phase of swing at which participants were tripped (*p* = .550), also did not differ between trip groups (Table 5). Moreover, no reactive balance measures (Table 5), fall incidence (*p* = .541; Table 5), or the stepping strategy over the obstacle (*p* > .999) differed between trip groups.

**Table 4:** Participant characteristics for the three trip groups. Values are means (standard deviation)

	Baseline (*n* = 17)	Post-Control (*n* = 5)	Post-PBT (*n* = 8)	ANOVA *p*-value
Female/Male	10/7	4/1	5/3	
Age (years)	70.8 (4.1)	69.4 (3.6)	66.6 (4.1)	.114
Height (m)	1.67 (0.11)	1.66 (0.04)	1.73 (0.09)	.253
Mass (kg)	76.3 (16.1)	77.6 (13.4)	81.6 (14.9)	.740
IPAQ (MET minutes per week)	3053 (2921)	2171 (1979)	3900 (4414)	.602
Falls in the past year	2 (12%)	0 (0%)	1 (13%)	.723^K^
Timed-up-and-go (s)	7.7 (1.2)	8.4 (0.5)	7.5 (0.9)	.081
POMA (0–28 [best])	26.7 (1.0)	27.0 (0.7)	27.4 (0.7)	.229
Falls Efficacy Scale (10 [best] - 100)	11.5 (2.7)	13.0 (6.7)	10.5 (1.4)	.448

*IPAQ* International Physical Activity Questionnaire, short form, *POMA* performance-oriented mobility assessment, ^K^ indicates Kruskal-Wallis Test was used

**Table 5:** Gait characteristics before tripping, and reactive balance measures in response to tripping, for the three trip groups. Values are means (standard deviation)

	Baseline (*n* = 17)	Post-Control (*n* = 5)	Post-PBT (*n* = 8)	ANOVA *p*-value
*Gait characteristics before tripping*				
Gait speed (m/s)	1.50 (0.11)	1.53 (0.11)	1.47 (0.09)	.093
Step length (%BH)	43.6 (2.5)	43.5 (2.7)	43.5 (2.9)	.988
Minimum toe clearance (mm)	19.1 (10.8)	15.0 (3.6)	20.4 (8.2)	.421
Required coefficient of friction	0.21 (0.02)	0.21 (0.02)	0.20 (0.02)	.550
Gait phase at trip onset (% swing)	60.8 (2.4)	62.3 (4.2)	61.7 (1.7)	.550
*Reactive balance in response to tripping*				
Trunk angle at TD (deg)	37.0 (9.6)	36.9 (2.2)	41.1 (3.7)	.059
Recovery step length (%BH)	54.3 (14.1)	53.7 (8.8)	64.1 (10.5)	.137
Minimum hip height (% standing hip)	88.9 (5.4)	86.9 (7.1)	90.5 (2.8)	.465
Margin of stability at TD (cm)	−39.2 (15.3)	−56.4 (24.7)	−31.7 (9.1)	.112

See Methods text for boundaries used to calculate margin of stability

*BH* body height, *TD* touchdown of first step over trip obstacle

## Discussion

The goal of this study was to evaluate the effects of PBT targeting slipping *and* tripping on laboratory-induced slips and trips among older adults. We hypothesized that participants completing PBT would exhibit a lower peak slip speed after a laboratory-induced slip, compared to participants before intervention or after a control intervention. This hypothesis was supported because post-PBT participants exhibited an 18% lower peak slip speed compared to post-control participants. We acknowledge the difficulty in interpreting the results that peak slip speed among post-PRT participants was lower compared to post-control participants, but not statistically significantly lower compared to baseline participants. However, we note that this was not a large-scale randomized clinical trial, and encourage a broader interpretation of the results including secondary measures that generally support better reactive balance and a lower incidence of falls among post-PBT participants compared to baseline participants. The lack of group differences in gait characteristics (Table 2) suggests that these improvements did not result from proactive gait adaptations in anticipation of a possible slip. We also hypothesized that participants completing PBT would exhibit a smaller trunk angle at touchdown of the first recovery step after a laboratory-induced trip, compared to participants before intervention or after a control intervention. This hypothesis was not supported because group differences in trunk angle at touchdown did not reach statistical significance. Similarly, group differences among all secondary outcome measures of reactive balance and fall incidence after laboratory-induced trips did not reach statistical significance. Overall, while accumulating evidence supports the beneficial effects of PBT simultaneously targeting slipping *or* tripping, and a small number of studies support PBT targeting slipping *and* tripping, the results of the present study indicate that additional PBT research is needed to achieve improvements on both slipping and tripping.

This study had several limitations that should be discussed. First, both post-PBT and post-control groups were exposed to the “other” perturbation prior to completing their assigned intervention, effectively making this perturbation part of each group’s intervention. It is unclear if or how this opposing perturbation influenced our results. Moreover, while this experimental design does not appear to be a threat to internal validity for comparisons between post-PBT participants and post-control participants since both groups experienced the same perturbation before intervention, it may present a threat to internal validity for comparisons between the baseline group and either post-PBT or post-control participants. This is because participants during post-intervention testing may have been anticipating the same perturbation as during baseline. Although we did not find any gait differences compared with the baseline group that suggest anticipation of a slip [41] or trip [42], we cannot rule out any unmeasured changes in gait or central set [43] prior to post-intervention perturbations. Such anticipation could have accentuated adverse trunk kinematics, for example, given that anticipation of and guarding against a slip (involving backward loss of balance and trunk rotation) could result in greater forward trunk rotation after a trip (involving forward loss of balance and trunk rotation). Second, the verbal instructions provided to participants prior to gait trials (and all lab-induced slips and trips) included a warning of a potential slip or trip. Again, this does not appear to be a threat to internal validity because all groups experienced the same instructions before all perturbations. However, it may have led to unmeasured changes in gait or central set and thus adversely affected external validity when compared to completely unexpected perturbations [44]. Third, the minimum amount of training required to elicit improvements in reactive balance 1 week after training (as investigated here) remains largely unknown. The amount of training included here was guided by previous PBT interventions among older adults that found improvements in reactive balance after one session of PBT involving slipping [45, 46], 1–12 sessions of PBT involving tripping [5, 15, 16], and one session of PBT involving slipping and tripping [21]. However, the amount of training required when targeting both slipping and tripping may be higher than when targeting one or the other. Fourth, all training and assessment sessions were conducted by a single investigator who was not blinded to participant group, which could have introduced bias into the results. Fifth, participants were screened to exclude individuals with health conditions that may increase their risk of injury during testing and training. It is therefore unclear how these results would generalize to individuals with such conditions, or other populations. Sixth, the study may have been underpowered due to estimating effect sizes from pilot-like studies that used modest sample sizes [47] and participant/methodological differences from the present study, and that sample sizes were planned for one-sided statistical tests even though two-sided tests were employed. Lastly, because PBT was individualized and pseudorandom in nature, its exact replication is not possible although conceptual replication could be conducted.

Our results related to slipping generally agreed with previous PBT studies targeting slipping and tripping. Similar to the present study, Bhatt et al. [18] reported improved COM state stability and increased minimum hip height after slipping as young adult participants were repeatedly exposed to laboratory-induced slips and trips during a single experimental session. Unlike the present study, though, they also reported proactive gait adaptations prior to slips and trips including a decrease in step length, an increase in toe clearance. While these changes may reduce the risk of slipping or tripping (which supports their findings of less frequent losses of balance with repeated slips and trips), it is unclear if these adaptations are retained and generalize to daily life where perturbations are less predictable [20]. Brodie et al. [19] also repeatedly exposed young adults to laboratory-induced slips and trips during a single experimental session, but employed a methodology that aimed to minimize any gait adjustments by controlling cadence and step length. Their results agree with the present study in that their primary outcome finding (reduced AP trunk sway after slipping) was associated with reduced slip speed and reduced COM distance/velocity relate to the slipping foot at touchdown of the first recover step. Okubo et al. [20] used the same training methodology as Brodie et al. [19], and their results agree with the present study in that reactive balance improvements of less posterior COM displacement during slip recovery, and decreased slip speed after repeated exposure to slips and trips. They also reported a proactive gait adaptation of increased toe height at mid-swing. Most recently, Okubo et al. [21] compared slip and trip reactive balance before and after the same methodology as Brodie et al. [19]. Their results generally agreed with the present study in that after training there were no gait adaptations, slip speed decreased, MOS and COM control at touchdown of the first recovery step improved, and fall incidence after slipping decreased from 44 to 0% among young adults, and from 29 to 14% among older adults.

Our results related to tripping showed both similarities and differences with prior studies. Unlike the present study, three prior studies reported that repeated slips and trips resulted in improved reactive balance after tripping including less forward COM position/velocity [18, 20], less trunk flexion [18, 19], greater hip height [18], and a longer initial recovery step [20]. Two of these studies reported proactive gait adaptations of an increase toe height [18, 20] and decrease in step length [18] that may have confounded reactive balance measures. Consistent with the present study, Okubo et al. [21] reported that repeated slips and trips resulted in no changes in gait, reactive balance after tripping, and fall incidence after tripping.

Multiple potential explanations exist for the improvement in reactive balance and fall incidence after slipping but not tripping in the present study. First, the backward loss of balance trials interspersed during trip training may have inadvertently contributed to slip training. This contribution would not only increase the volume of slip training, but also provide two differing training modalities that may improve generalizability to slips while walking. Second, because trip training involved standing perturbations on a treadmill, it could be considered less task-specific to our assessment involving tripping while walking compared to slip training and assessment that both involved slipping while walking. Though the treadmill trip training employed here can reduce falls inside [15, 48] and outside [16] the lab, less task specificity than the slip training may have resulted in a smaller effect size and thus required a larger training volume or sample size. Third, it remains unclear whether the training dosage needed to elicit reactive balance improvements that are both lasting and transferrable to daily life differs between slipping and tripping, or between training modalities (e.g. with our without a treadmill) [49]. Fourth, training interference, which can occur when two trained tasks have conflicting requirements [50], may require more training when PBT targets both slipping and tripping to achieve equivalent improvements when targeting slipping or tripping. For example, improved reactive balance after tripping is associated with more *posterior* COM position relative to the stepping foot, while improved reactive balance after slipping is associated with more *anterior* COM position relative to the slipping foot [19]. Lastly, we may have overestimated the effect size of PBT on trip-related measures by using prior studies employing different PBT modalities.

Three prior PBT studies targeting slipping and tripping measured trip-induced fall incidence [18, 20, 21]. All three reported no trip-induced falls during training or testing. In contrast, all three of these studies reported at least one slip-induced fall during training or testing (albeit the total number of slip-induced falls across all three studies was low). This inconsistency between trips and slips seems noteworthy, although the potential explanation is unclear. It could be due to a greater difficulty in recovering from these lab-induced slips than trips, or in the methodology used to distinguish between falls and recoveries. Participant characteristics could also have a role, in that only Okubo et al. [21] included older adults (who generally exhibit greater difficulty recovering balance after tripping), while Bhatt et al. [18] and Okubo et al. [20] included only young adults. The ability to induce and identify PBT-induced improvements may be enhanced by ensuring the training and assessments are sufficiently challenging when balanced with participant physical capacity.

The PBT methods employed in the present study to target slips and trips appeared to be well-tolerated by healthy community-dwelling older adults. Okubo et al. [21] reported a non-trivial dropout rate and elevated anxiety levels among older adults during one session of repeated slips and trips while walking. Although anxiety levels were not recorded in the present study, all participants completed all four sessions of PBT, suggesting a high level of participant acceptance using the selected training modalities. The general lack of dropouts may be related to an overall clearer expectation of type of perturbation during slip or trip training given that Okubo et al. [21] reported both dropout and anxiety increased with unpredictability of the perturbations (even though the trip training employed here included a small number of unexpected backward loss of balance perturbations). It is unclear, however, how a greater participant expectation of the type of perturbation (i.e. slip or trip) influences the efficacy of PBT. However, reducing participant anxiety during training to maximize participation and adherence, such as through training individualization, is a key consideration for clinical implementation [51] and community adoption [52].

## Conclusions

PBT targeting slipping and tripping improved reactive balance and fall incidence after laboratory-induced slips among older adults. Improvements were not observed after laboratory-induced trips, and may have resulted from differences in dosage and specificity between slip and trip training.

## Supplementary information

**Additional file 1.**

## Data Availability

The datasets used and/or analyzed during the current study are available from the corresponding author on reasonable request.

## Abbreviations

AP: Anteroposterior
PBT: Perturbation-based balance training
COM: Center of mass
