# Supplementary material for: Perturbation-based balance training targeting both slip- and trip-induced falls among older adults: a randomized controlled trial
Source: BMC Geriatr. 2020 Jun 12;20:205. doi: 10.1186/s12877-020-01605-9 (PMC7291462; doi:10.1186/s12877-020-01605-9)
Supplement: Supplementary file 1 — Additional file 1. [file 12877_2020_1605_MOESM1_ESM.pdf]

Supplemental Statistical Results and Results Plots for:
Perturbation-based Balance Training Targeting Both Slip- and Trip-Induced Falls Among Older Adults: a Randomized Controlled Trial

Table S1 - Additional statistical results for participant characteristics among the three slip groups.

	f -statistic	df_{num}	df_{den}	*p* -value	eta squared
Age (years)	1.877	2	9.965	0.203	0.119
Height (m)	0.333	2	10.432	0.724	0.027
Mass (kg)	0.881	2	11.162	0.441	0.072
IPAQ (MET minutes per week)	1.536	2	8.862	0.268	0.164
Timed-up-and-go (s)	0.924	2	11.188	0.425	0.072
POMA (0-28 [best])	0.944	2	12.501	0.415	0.056
Falls efficacy scale (10 [best] - 100)	k	k	k	0.332	k

Note: IPAQ = International Physical Activity Questionnaire, short form; POMA = performance-oriented mobility assessment; k indicates Kruskal-Wallis Test was used.

Table S2 - Additional statistical results for gait characteristics and reactive balance measures among the three slip groups.

	f -statistic	df_{num}	df_{den}	*p* -value	eta squared
Gait Characteristics Before Slipping					
Gait speed (m/s)	1.293	2	10.582	0.314	0.097
Step length (%BH)	0.679	2	9.739	0.530	0.052
Minimum toe clearance (mm)	1.719	2	9.564	0.230	0.143
Required coefficient of friction	0.287	2	9.771	0.757	0.025
Reactive Balance in Response to Slipping					
Peak slip speed (m/s)	4.255	2	11.599	**0.041**	0.266
Slip distance (cm)	1.161	2	11.946	0.346	0.087
Non-slipping toe to COM at TD (%BH)	0.742	2	11.262	0.498	0.054
Minimum hip height (%)	4.706	2	10.206	**0.036**	0.273
Margin of stability at TD (cm)	5.299	2	10.158	**0.027**	0.266
vCOM relative to BOS at TD (m/s)	10.272	2	10.232	**0.004**	0.416

Note: Bold indicates statistically significant main effect of slip group. Minimum hip height is expressed as a percentage of standing hip height. BH = body height; COM = center of mass; TD = touch down of non-slipping foot after reactive stepping; vCOM = anterior-posterior velocity of the COM; BOS = base of support.

Table S3 - Additional statistical results for participant characteristics among the three trip groups.

	f-statistic	df_{num}	df_{den}	*p*-value	eta squared
Age (years)	2.689	2	10.577	0.114	0.180
Height (m)	1.510	2	14.829	0.253	0.072
Mass (kg)	0.310	2	10.925	0.740	0.023
IPAQ (MET minutes per week)	0.531	2	11.470	0.602	0.032
Timed-up-and-go (s)	2.998	2	14.632	0.081	0.077
POMA (0-28 [best])	1.677	2	11.712	0.229	0.103
Falls Efficacy Scale (10 [best] - 100)	0.876	2	9.281	0.448	0.058

Note: IPAQ = International Physical Activity Questionnaire, short form; MET = metabolic equivalent of task; POMA = performance-oriented mobility assessment.

Table S4 - Additional statistical results for gait characteristics and reactive balance measures among the three trip groups.

	f-statistic	df_{num}	df_{den}	*p*-value	eta squared
Gait Characteristics Before Tripping					
Gait speed (m/s)	0.696	2	9.743	0.522	0.048
Step length (%BH)	2.260	2	9.605	0.157	0.183
Minimum toe clearance (mm)	0.047	2	10.565	0.955	0.004
Required coefficient of friction	3.702	2	12.806	0.054	0.154
Gait phase at trip onset (% swing)	0.637	2	9.392	0.550	0.053
Reactive Balance in Response to Tripping					
Trunk angle at TD (deg)	3.362	2	16.597	0.059	0.058
Recovery step length (%BH)	2.343	2	12.424	0.137	0.119
Minimum hip height (% standing hip)	0.828	2	9.815	0.465	0.053
Margin of stability at TD (cm)	2.768	2	9.563	0.113	0.219

Note: BH = body height; TD = touch down of first step over trip obstacle. A less negative margin of stability indicates the center of mass is outside of (and posterior to) the base of support, but closer to the boundary of the base of support.

Gait characteristics before slipping

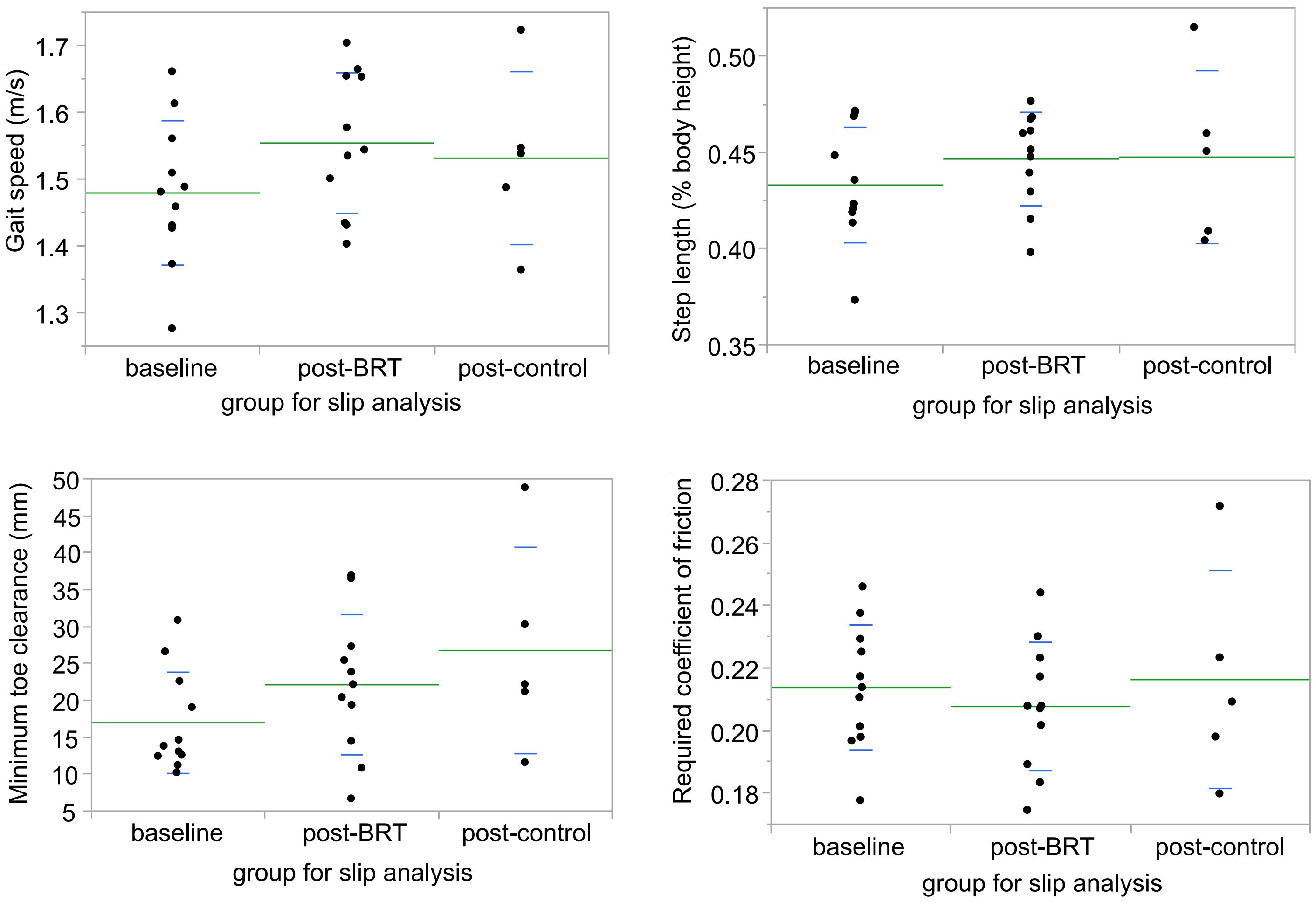

Note: Wide green horizontal lines indicate group means. Narrow blue horizontal lines indicate standard deviations.

Gait characteristics before tripping

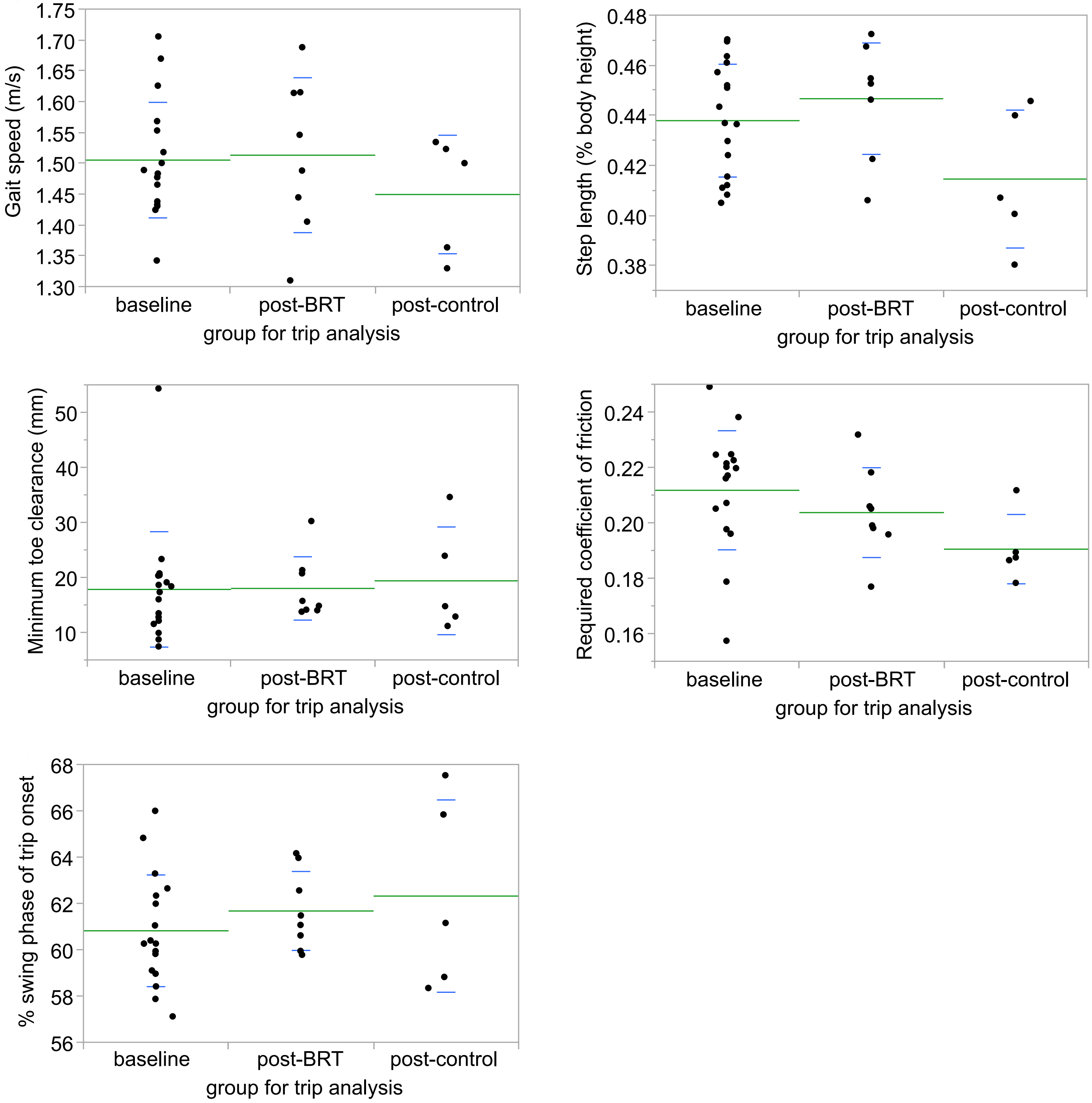

Note: Wide green horizontal lines indicate group means. Narrow blue horizontal lines indicate standard deviations.

Participant characteristics for the three slipping groups

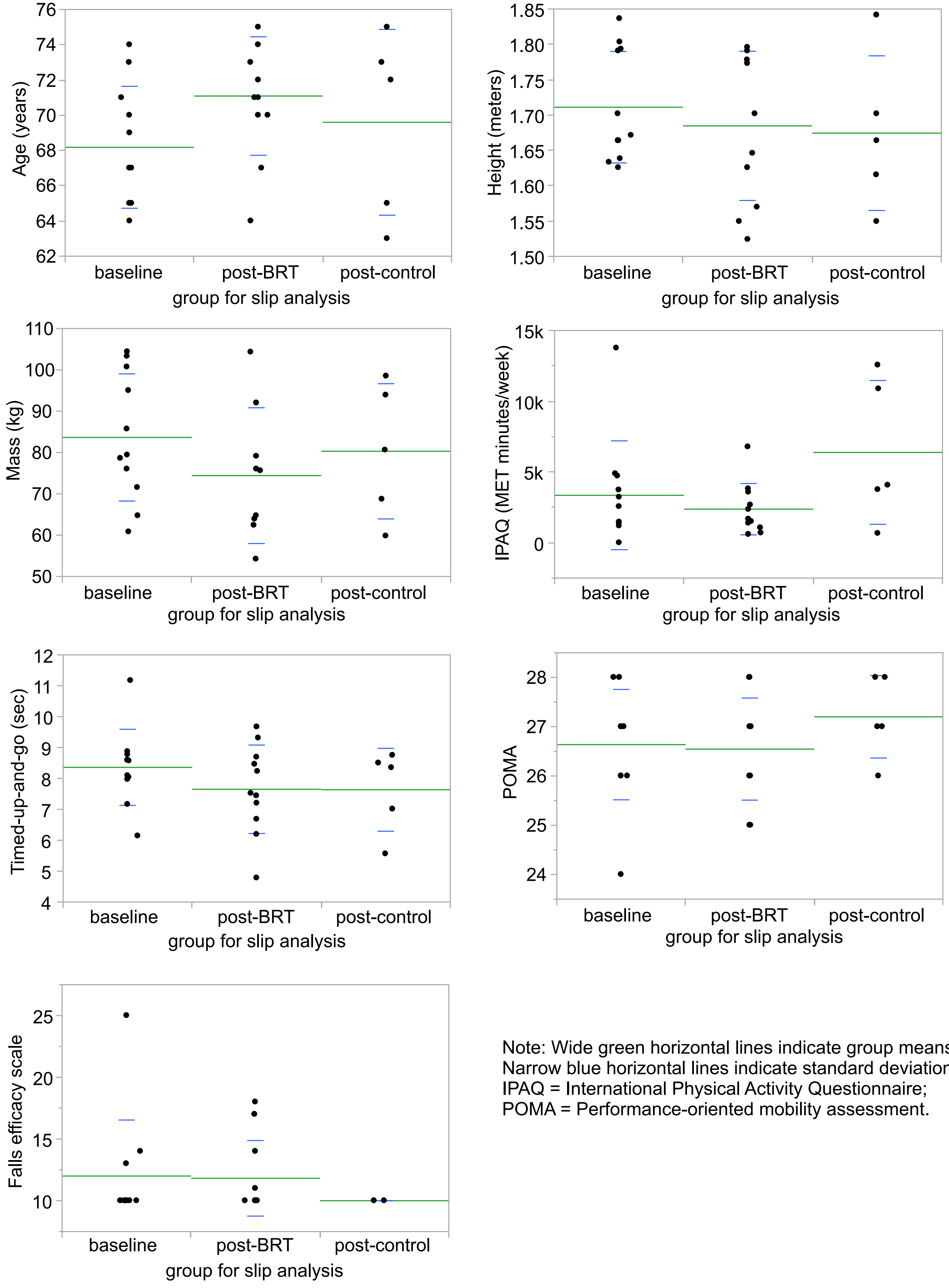

Note: Wide green horizontal lines indicate group means.
Narrow blue horizontal lines indicate standard deviations.
IPAQ = International Physical Activity Questionnaire;
POMA = Performance-oriented mobility assessment.

Participant characteristics for the three trip groups

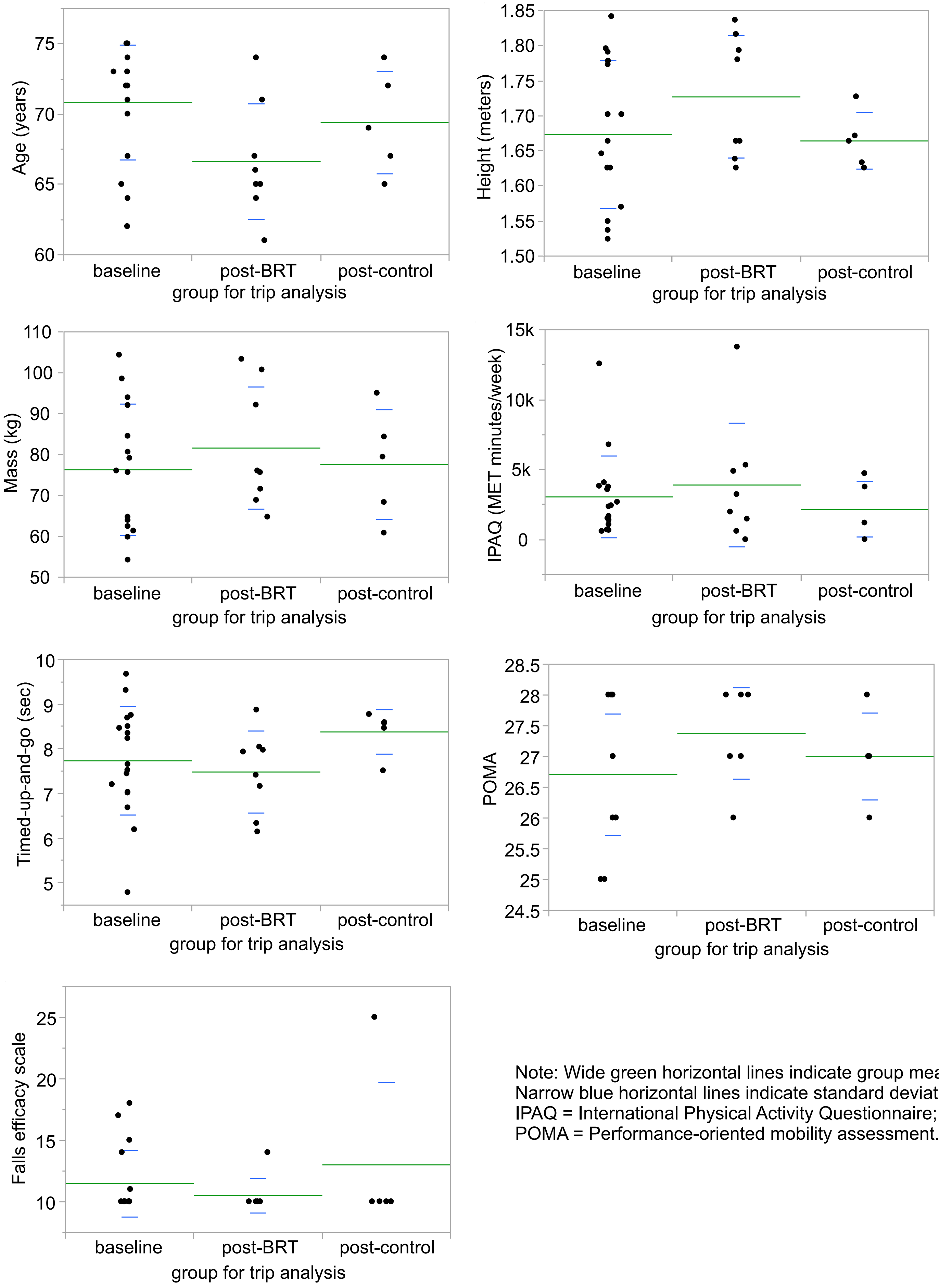

Note: Wide green horizontal lines indicate group means.
Narrow blue horizontal lines indicate standard deviations.
IPAQ = International Physical Activity Questionnaire;
POMA = Performance-oriented mobility assessment.

Reactive balance measures after slipping

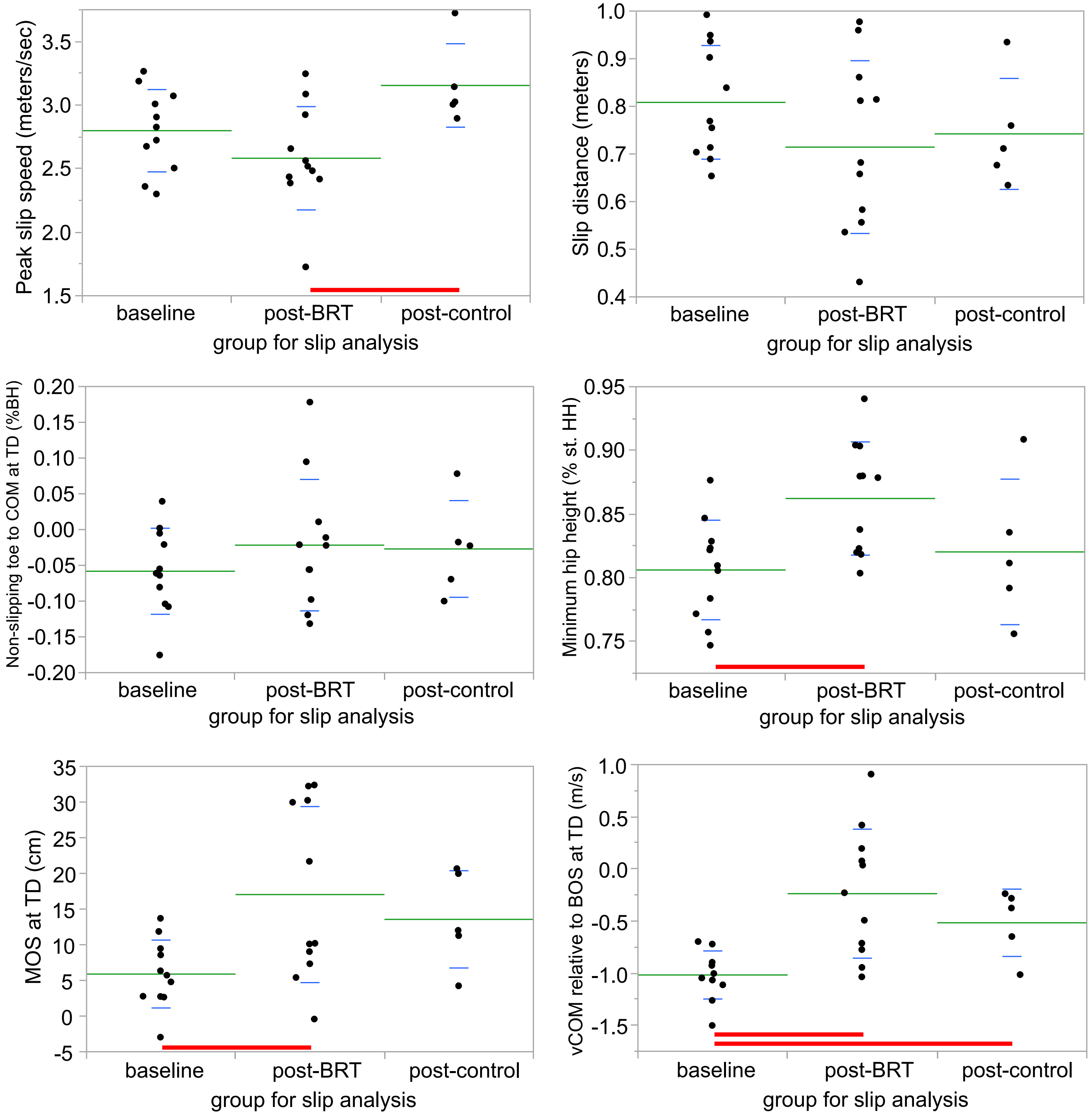

Note: Wide green horizontal lines indicate group means. Narrow blue horizontal lines indicate standard deviations. Red lines indicate statistical difference between groups connected at endpoints. COM = center of mass; TD = touchdown of initial balance recovery step; BH = body height; st. HH = standing hip height; MOS = margin of stability; vCOM = velocity of center of mass; BOS = base of support.

Reactive balance measures after tripping

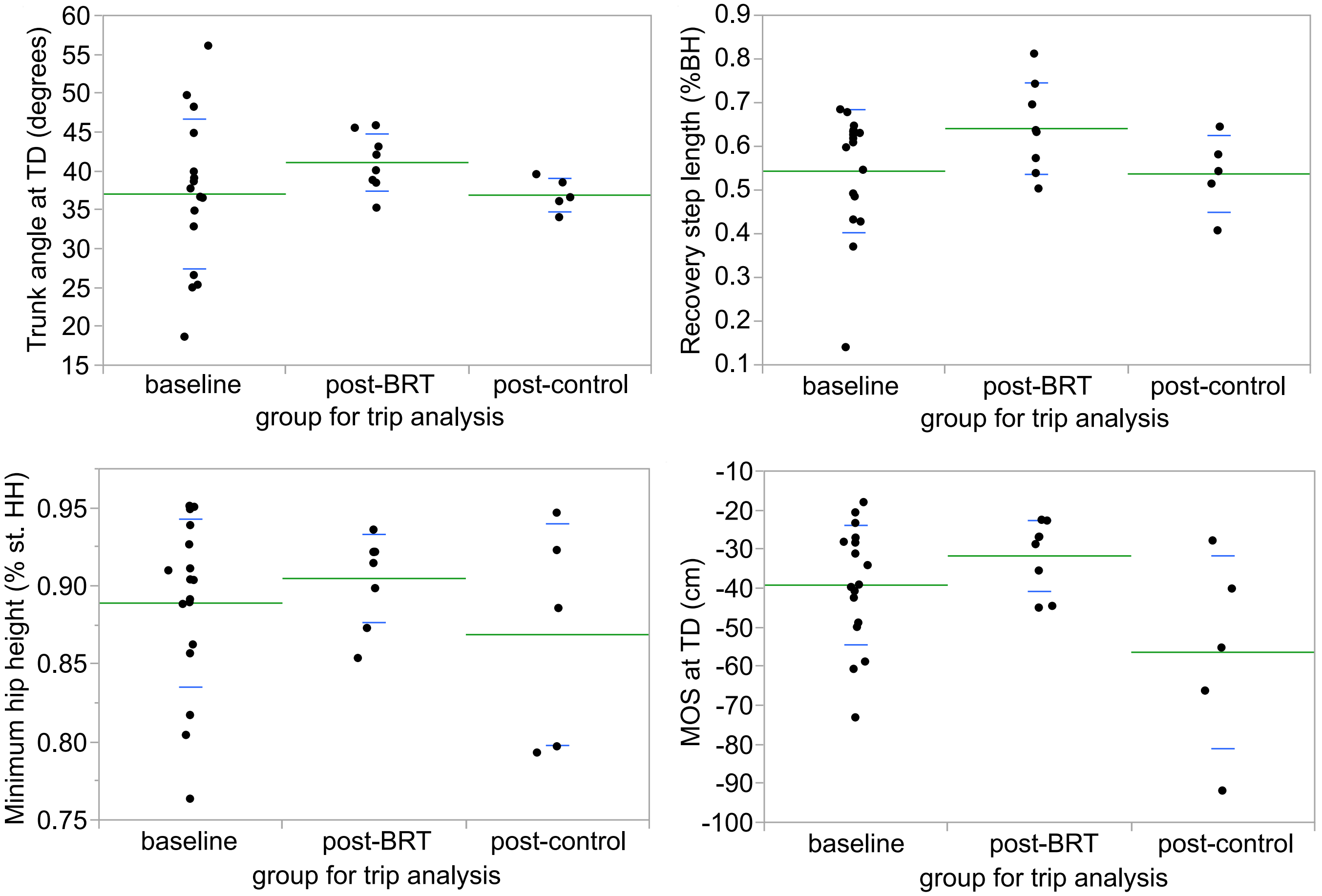

Note: Wide green horizontal lines indicate group means. Narrow blue horizontal lines indicate standard deviations. TD = touchdown of initial balance recovery step; BH = body height; st. HH = standing hip height; MOS = margin of stability.
